# Human Infection Challenge Studies: a Test for the Social Value Criterion of Research Ethics

**DOI:** 10.1128/mSphere.00669-20

**Published:** 2020-07-15

**Authors:** Nicholas G. Evans

**Affiliations:** a Department of Philosophy, University of Massachusetts Lowell, Lowell, Massachusetts, USA; University of Michigan-Ann Arbor

**Keywords:** COVID-19, challenge studies, coronavirus, global health, research ethics

## Abstract

Human infection challenge studies involving the intentional infection of research participants with a disease-causing agent have recently been suggested as a means to speed up the search for a vaccine for the ongoing coronavirus disease 2019 (COVID-19) outbreak. Calls for challenge studies, however, rely on the expected social value of these studies. This value represents more than the simple possibility that a successful study will lead to the rapid development and dissemination of vaccines but also some expectation that this will actually occur. I show how this expectation may not be realistic in the current political moment and offer potential ways to make sure that any challenge trials that arise actually achieve their goals.

## Abstract (precis)

Human infection challenge studies involving the intentional infection of research participants with a disease-causing agent have recently been suggested as a means to speed up the search for a vaccine for the ongoing coronavirus disease 2019 (COVID-19) outbreak. Calls for challenge studies, however, rely on the expected social value of these studies. This value represents more than the simple possibility that a successful study will lead to the rapid development and dissemination of vaccines but also some expectation that this will actually occur.

## PERSPECTIVE

The ongoing coronavirus disease 2019 (COVID-19) outbreak has caused more than 10 million confirmed cases of illness and over 500,000 deaths worldwide. Countering such a pandemic, particularly given its global scope and a history of political missteps in responding to the virus in its early stages, will arguably require a vaccine. Human infection challenge studies (here referred to simply as “challenge studies”) involve the intentional infection of research participants with a disease-causing agent. It has been suggested that challenge studies could accelerate the development of a vaccine, with the intention of shortening the outbreak and limiting the loss of life (1–3). However, intentional infection of humans with a disease-causing agent that has a high overall mortality rate, and for which no current therapeutic measures exist, has generated considerable ethical debate.

The purpose of this essay is not to argue whether challenge studies are ethically justified in principle (or, put another way, in the general sense). This is, or should be, uncontroversial: the ethical guidelines for challenge studies were established almost 20 years ago (4), and challenge studies have been conducted in justifiable ways with infectious diseases for longer (5–7). Nor is it to argue whether challenge studies are ethically justified, in principle, in the case of COVID-19. Rather, I examine whether the central basis of justification of this kind of study—its social value—can be justified in practice and, in particular, in the uncertain and volatile times in which COVID-19 has emerged.

## THE SOCIAL VALUE OF RESEARCH

A central justification for challenge studies is the social benefit they might entail. Challenge studies performed with a small number of robustly informed, consenting, compensated, and medically supported volunteers may be justified if they cut the time to the development and deployment of an efficacious vaccine for COVID-19. This saved time, in the context of the ongoing pandemic, could result in fewer deaths—perhaps many thousands fewer—than would occur without such studies (2).

This benefit, similar to the benefit posited when challenge studies were discussed in the context of the Zika virus disease outbreak that emerged in 2015, would ostensibly be global (8). Recent commentary on challenge studies in the context of COVID-19 has assessed the value of a vaccine for this disease as greater than that of a cure for HIV/AIDS (9). Even in cases where challenge studies might not be directly testing a promising vaccine candidate, but might be performed to clarify the dynamics of infection or viral pathogenesis, among other important issues, the ultimate referent of the value of this research is a vaccine to end the global pandemic (2).

The magnitude of this benefit, however, will likely not be as great as hoped given the current climate into which challenge studies emerge. This is significant: we know that not all benefits justify challenge studies. In the case of Zika virus disease, initial surveys of the ethics of challenge studies in December 2016 led to the conclusion that a lack of (i) a strong argument that a challenge study would accelerate vaccine development or (ii) an indication that field trials of vaccines would be prohibitively difficult to conduct spoke against pursuing a challenge study at the time (10). What it suggests is that, if not a minimal benefit, a minimal risk-benefit ratio is needed to justify challenge studies.

The bigger problem that arises in the context of COVID-19 is that vaccines are not vaccinations. It is doubtful that anyone is interested in merely possessing a COVID-19 vaccine; the aim is to end the pandemic. Moreover, when discussing cures or vaccines for pandemic diseases, it is not simply that we are interested in the possibility of deploying a vaccine or in vaccinating a very small number of people. We want to have some confidence that this would actually come to pass, at scale, and in the world we live in (11).

## HOW CONFIDENT SHOULD WE BE?

Recent writing on challenge studies noted that hurdles to realizing the social value requirement described above often come in two forms. First, candidate vaccines resulting from challenge studies need to make it to the people who need vaccination. Second, communities that may be asked to participate in challenge studies, or that may be disproportionately impacted by decisions (including those arising from challenge i) in vaccine design and distribution, must be engaged (2, 3).

What confidence should we have that we are on the right path to surmounting these challenges as we make the assessment of when, if, or how to pursue challenge studies? Concerning the first hurdle, the situation is not rosy. While alliances exist such as the Coalition for Epidemic Preparedness Innovations (CEPI) and Gavi, the Vaccine Alliance, these are not necessarily equipped for the level of mobilization required for a COVID-19 vaccination campaign (12). The World Health Organization is attempting to take a leading role in ensuring that challenge studies are feasible and socially valuable, but such efforts may be severely undermined by the United States’ current plans to withdraw its membership from the agency (13). Yet even if it were not to do so, instruments such as the Pandemic Influenza Preparedness framework—arguably the benchmark for ensuring access to vaccines during a disease pandemic—have been criticized for lacking the commitment by states necessary to meet demand (14), throwing into question the possibility of access to and sharing of a COVID-19 vaccine.

The actions of state and substate actors give us reason to fear that the political will to distribute an efficacious vaccine is limited. Internationally, assertions of sovereignty by the United Kingdom and the United States in claiming priority over access to a vaccine have been documented in the course of this pandemic (15). Domestically in the United States, the situation is likewise perilous. Previous work on challenge studies has suggested the U.S. Government might use the “march-in” provisions of the Bayh-Dole Act (2), where the funding agency asserts rights to expanded licensing (i.e., beyond that reserved for patent holders) for innovations developed with public funds. Yet in the 40 years since Bayh-Dole was passed, those provisions have never been utilized (16). It is not impossible that march-in rights might be used during COVID-19, but history speaks strongly against it. Highly inequitable, ultimately ineffective vaccination programs may fail to achieve their goals because of political will rather than scientific roadblocks.

The second hurdle may be even more daunting. The United States, in particular but not exclusively, is experiencing a resurgence in conspiracy theories and extremist behavior in the context of COVID-19. These conspiracy theories are often directed against actors who are, for better or worse, leaders in developing and deploying vaccines: conspiracy theories concerning The Bill and Melinda Gates Foundation; the United Nations and World Health Organization; and the U.S. National Institutes of Health are rampant. Some may verge on the unbelievable, such as the conspiracy theory that gain-of-function research conducted on severe acute respiratory syndrome (SARS)-like coronaviruses in 2015 is connected to the emergence of COVID-19 that made it to British tabloids (17), or the conspiracy that ongoing measures to increase social distancing represent a prologue to forced vaccination campaigns intended to cause global depopulation popular with conspiracy theorists in the United States (18). But others, even if false in this moment, are grounded in the dark history of the life sciences. The ongoing concern that developing nations are simply treated as laboratories by developed nations to test dangerous drugs is absolutely grounded in the history of exploitative clinical trials not just abroad but in the United States itself. The latter conspiracy theory has made it as far as prominent right-wing commentators in the United States (19), which arguably means that it is nearing prime-time dissemination.

## SOCIAL VALUE IN EXTRAORDINARY TIMES

The capacity of these hurdles to derail vaccine efforts should not be underestimated. The last of these was enough to lead a U.S. official to claim that vaccination in the US could fail to contain the virus following a CNN poll that announced that one-third of Americans surveyed would not try to get vaccinated against COVID-19 (20). Another poll showed 20% of all participants responding “no” to plans to get a COVID-19 vaccine, rising to 40% for Black respondents (21). This calls the expected actual social value of a challenge study into question. Many of these issues are part of a broader array of challenges facing vaccine development. But some, particularly community engagement challenges, may become ever more acute as the prospect of intentional infection with COVID-19 is advanced. The narrative of intentionally infecting a participant with a disease for the purpose of giving them a vaccine is homologous to existing and widespread conspiracies, some grounded in the long historical injustices visited on vulnerable communities. Because challenge studies require a particularly favorable risk-benefit ratio or even minimal expected social benefit in virtue of the risk they entail, those studies are left in an ethically fraught position.

This does not mean, however, that challenge studies are inherently unethical. Severe doubts about the expected social value of challenge studies exist in this moment. One response is to modify the way that we conduct clinical trials. This modification would see vaccine trials approved in concert with access and benefit sharing mechanisms and with the firm commitment of funds to ensure sustainable distribution prior to commencing challenge studies. There is still time to do this, given that many vaccine candidates are in the early stages of development.

It also means conceiving of public engagement in the broadest possible terms and engaging communities early and on their own terms. This would require mobilization of resources to engage the many thousands of local communities that might be affected either by inequities in vaccine distribution or by both real and fantastical fears about challenge studies and vaccines. This engagement is not merely for education but also to determine and respond to the needs of those communities, ideally with their participation. Governments could do this, but given that antivaccination sentiment can often be derived from broader distrust of government and industry (22), other stakeholders may need to engage communities directly.

This is no small challenge—it conceivably represents a bigger challenge than the technical task of finding a vaccine. But in order to justify the demand for resources and the risk of challenge studies, it is necessary. Without it, the social benefit of the rapid search for a COVID-19 vaccine will remain unrealized.
